# Impact of genome duplications in drought tolerance and distribution of the diploid-tetraploid *Jasione maritima*

**DOI:** 10.3389/fpls.2023.1144678

**Published:** 2023-02-23

**Authors:** Helena Castro, Maria Celeste Dias, Mariana Castro, João Loureiro, Sílvia Castro

**Affiliations:** Centre for Functional Ecology, Department of Life Sciences, University of Coimbra, Calçada Martim de Freitas, Coimbra, Portugal

**Keywords:** diploid-tetraploid complex, neopolyploid, parapatric contact-zone, polyploidy, water stress

## Abstract

Polyploidy has important ecological effects, including ploidy-mediated effects on morphology, breeding system and ecological tolerances. However, there is still little comprehensive research available to test its adaptive significance and its role in driving distributional patterns. This work aimed to assess the contribution of genome duplications to ecological divergence using an experimental approach with the diploid-tetraploid *Jasione maritima* polyploid complex. We explored if individuals with different ploidy differ in their tolerance to water deficit and if this may contribute to explaining the distribution patterns along a latitudinal gradient in the northwest Iberian Peninsula. For that, we used three cytogenetic entities: diploids and established tetraploids collected in natural populations along a latitudinal gradient, and neotetraploids synthesized from diploid populations after treatments with colchicine. Thirty plants from each of the nine populations were grown under controlled conditions with half randomly assigned to the water deficit treatment, and half used as control. We determined experimental plants’ response by measuring fitness-related parameters, such as above and belowground biomass, plant water status, photosynthetic efficiency and pigments, membrane stability, antioxidant capacity and sugars content. Our data shows that biomass, chlorophyll content, photochemical quenching (qP) and non-photochemical quenching (NPQ) in neotetraploids and established tetraploids were significantly higher than in diploids and that these differences could be attributed to genome duplications. In response to the water deficit, diploids seem to use a strategy of avoidance, whereas tetraploids seem to employ the strategy of tolerance to overcome water deficit stress, which appears equally efficient. Additionally, we did not observe a response pattern along the latitudinal gradient of the distributional range of the *J. maritima* complex. The results indicate that the response to water deficit is population dependent. Further studies are necessary to understand the role of ploidy in explaining the distribution patterns of the *J. maritima* complex.

## 1 Introduction

Despite the importance of the rapid ecological effects of polyploidy, there is still little comprehensive research available to test its adaptive significance and role in driving different distributional patterns. Polyploidization can cause changes in morphology and physiology, potentially conferring different environmental adaptations and tolerances to neopolyploids, and in gene redundancy, which shields (neo)polyploids from harmful effects of mutations and grants the ability to diversify gene function (Comai, 2005; Ramsey, 2011; del Pozo and Ramirez-Parra, 2015), possibly facilitating adaptive variation (Parisod et al., 2010). The changes after polyploidization can, thus, enable the new cytotypes to respond differently to the surrounding biotic and abiotic environment.

Physiological traits of polyploids such as growth rate, photosynthetic activity, stomata size and function, secondary metabolites production, photosynthetic pigment content and drought tolerance have been shown to differ from those of related lower ploidy individuals (Maherali et al., 2009; Liu et al., 2011; Münzbergová and Haisel, 2018; van de Peer et al., 2021). Tetraploids tend to have higher contents of chlorophyll and carotenoids (*e.g.*, Cao et al., 2018; Mao et al., 2018) and sugars (Mao et al., 2018) when compared to diploids, which may confer higher protection against excess light energy. Tetraploids also tend to have larger but fewer stomata resulting in lower transpiration rates, as shown for several plant species [*e.g.*, *Jasione maritima* (Siopa et al., 2020), *Arabidopsis* (Del Pozo and Ramirez-Parra, 2014), *Solidago gigantea* (Walczyk and Hersch-Green, 2022)]. Additionally, tetraploids tend to be bigger and have higher fitness than diploids, which may facilitate their establishment (Ramsey, 2011; del Pozo and Ramirez-Parra, 2015) and can have larger leaf thickness (van Laere et al., 2011) or epidermis thickness and pubescence (Li et al., 1996, Li et al., 2009), which may result in higher tolerance to stressful factors. Finally, tetraploids tend to have a more efficient antioxidant system, eliminating reactive oxygen species (ROS) produced under stressful conditions and enabling them to avoid photo-oxidation under these conditions (Del Pozo and Ramirez-Parra, 2014; Pintó-Marijuan and Munné-Bosch, 2014; Tan et al., 2015; Kong et al., 2017). However, there are also exceptions, and several studies report no differences between diploids and polyploids [*e.g.*, *Ranunculus adoneus* (Baack and Stanton, 2005); *Aster amellus*, (Münzbergová, 2007); *Senecio carniolicus* (Hülber et al., 2011); *Allium oleraceum* (Fialová and Duchoslav, 2014); *Chamerion angustifolium* (Thompson et al., 2015)) or their results point to population or variety dependent responses (Garbutt and Bazzaz, 1983; van Laere et al., 2011; Eliášová and Münzbergová, 2017; Münzbergová and Haisel, 2018).

The changes in developmental and physiological traits are expected to interfere with the ability of the cytotypes to respond to biotic and abiotic environments and, consequently, shape their distribution through the landscape (Levin, 2002; Hao et al., 2013). Polyploids usually occupy drier habitats than their lower ploidy parentals (Maherali et al., 2009; Manzaneda et al., 2012), although several studies comparing the response of diploids and polyploids to water dificit showed results that range from no differences to polyploids being less sensitive than diploids or vice versa (Li et al., 1996; Levin, 2002; Buggs and Pannell, 2007; Maherali et al., 2009; Jiang et al., 2022). For example, diploids of *Mercurialis annua* showed higher water use efficiency, lower transpiration and higher photosynthetic rate when compared to hexaploids, indicating that diploids were more tolerant to drought than the hexaploids. However, no significant differences were found in plant biomass between cytotypes (Buggs and Pannell, 2007). On the contrary, diploids of *Betula pyrifera* suffered a more substantial decline in photosynthesis than polyploids when exposed to water stress, indicating that diploids have a higher sensitivity to water stress than pentaploids and hexaploids (Li et al., 1996). Tetraploids of *Chamerium angustifolium* showed more robust performance than diploids under water stress (Maherali et al., 2009), and tetraploids of *Citrus wilsonii* showed less water loss and cellular damage than diploids under water stress, indicating that tetraploids have a higher tolerance to water stress (Jiang et al., 2022). Understanding the changes produced after polyploidization and its impacts under stressful conditions such as water deficit may enable us to understand the success of polyploid lineages and their current distribution patterns.

*Jasione maritima* (Duby) Merino is a dune species with a parapatric distribution of diploid (2*x* var. *maritima*) and tetraploid (4*x* var. *maritima* and 4*x* var. *sabularia*) populations occurring along a latitudinal gradient in the northwest Iberian Peninsula. Diploids occur in the wetter north of Galicia from Ferrol to Lariño, Spain (var. *maritima*), and tetraploids (var. *maritima* and var. *sabularia*) from Lariño southern to Aveiro, Portugal, which is marked by a drier and hotter environment (Castro et al., 2020). This pattern suggests that tetraploids may have some advantage compared with their diploid counterparts, enabling them to colonize Southern areas. Additionally, niche modelling analyses suggest that the environmental niche of tetraploid var. *sabularia* is distinguishable from both diploid and tetraploid var. *maritima* cytotype niches (Castro et al., 2020). Tetraploid var. *sabularia* colonized areas facing S-SW, which are hotter and slightly drier environments than those occupied by var. *maritima* (Castro et al., 2020). Indeed, morphological studies show differences in a few plant traits between northern diploid var. *maritima* and southern tetraploids var. *maritima* and var. *sabularia*, as well as between tetraploid varieties, namely in the plant ramification and inflorescence size (Rubido-Bará et al., 2010). Competitive ability experiments using var. maritima cytotypes showed that neotetraploids are the best competitor. Still, results also suggest a gradient in root length, with tetraploids investing more in belowground biomass (Castro et al., under review). The higher investment in roots by tetraploids may be linked with resistance to drought and may explain different fitness successes between diploids and tetraploids across their distribution range. This hypothesis can be formally tested using drought experiments under controlled conditions. Furthermore, given that neotetraploids have been successfully synthesized from diploid *J. maritima* (Castro et al., 2018), including them in the comparisons will allow for disentangling the role of genome duplications per se from the natural selection that occurred after polyploidization.

This work aimed to assess the contribution of genome duplications to ecological divergence, particularly tolerance to water deficit, using an experimental approach with the diploid-tetraploid *J. maritima* polyploid complex. We explored if diploids, neotetraploids and established tetraploids differed in their tolerance to water deficit and posed the following specific questions: 1) do diploids, neotetraploids and established tetraploids differ in morphological and physiological traits? We hypothesized that genome duplications produce changes in the newly arisen tetraploids; alternatively, differences might have arisen through selection after the emergence of the polyploid. The morphological and physiological comparisons between diploids, neotetraploids, and established tetraploids growing under controlled conditions will enable disentangling of these two processes. 2) Do differences between cytotypes increase the ability to cope with exposure to water deficit? We hypothesized that tetraploids have higher tolerance to water deficit and consequently higher fitness under stressful conditions than diploids explaining current distributional patterns where tetraploids occupy southern locations marked by higher temperatures and more extended water deficit periods; in contrast, diploids occupy northern locations with higher environmental moisture. If different strategies between diploids, neotetraploids and established tetraploids are found, we may explain (some of) the factors involved with the successful establishment of neotetraploids, which is crucial to understand the adaptive value of polyploidy and its widespread occurrence in nature.

## 2 Methods

### 2.1 Plant material

We used three cytogenetic entities: diploids and established tetraploids collected in natural populations along a latitudinal gradient that spans from Aveiro (Portugal) in the south, to the north of Galicia (Spain; **Table 1**), and neotetraploids synthesized from the southernmost diploid population after treatments with colchicine (Castro et al., 2018). Seeds were collected in three diploid populations in the north of Galicia (Spain) from Ferrol to Lariño and five tetraploid populations from Lariño, Galicia, to Aveiro, Portugal (**Table 1**). The seeds from the neotetraploids were collected from hand-crossed between synthetic neotetraploid plants, which were obtained in the laboratory through the treatment with colchicine of seeds collected from the diploid population in the contact zone, as described by (Castro et al., 2018), thereby potentially minimizing the effect of the exposition to colchicine (Husband et al., 2008).

**Table 1:** Location and DNA ploidy level (2*x*-diploid, 4*x*-tetraploid) of the natural *Jasione maritima* populations.

Populations	*Jasione maritima* variety	DNA Ploidy level	Latitude	Longitude
MS013- Lage, Soesto, La Coruña, Spain	var. *maritima*.	2*x*	43.21240	-9.02343
SC077- Fisterra, Afora beach, La Coruña, Spain	var. *maritima*.	2*x*	42.90851	-9.27328
SC073- Lariño, La Coruña, Spain*	var. *maritima*.	2*x*	42.77103	-9.12227
SC072- Ventim, Abelheira, La Coruña, Spain*	var. *maritima*.	4*x*	42.79917	-9.02685
SC080- Basoña, La Coruña, Spain	var. *maritima*.	4*x*	42.61898	-9.05401
SC116- Liméns, Pontevedra, Spain	var. *maritima*.	4*x*	42.26023	-8.81370
MC220- Anha, Viana do Castelo, Portugal	var. sabularia	4*x*	41.66749	-8.82249
MC217- Sisto, Esmoriz, Portugal	var. sabularia	4*x*	40.98698	-8.64463

The * indicates the populations from the contact zone between diploids and tetraploids of var. maritima. The population highlighted in bold was the natural diploid population used in neotetraploids synthesis.

Ten days before the start of the water stress experiment in the greenhouse, seeds from the nine populations (three diploid, five tetraploid and one neotetraploid) were placed in individual Petri dishes with filter paper moistened with distilled water and stored at 4°C for 5 days. This cold treatment enabled the synchronization of seed germination (Castro et al., 2018) and ensured that all the seedlings had similar sizes at the time of transplant to pots. After that period, Petri dishes were transferred to a climatic chamber at 24°C with a 16h:8h (light:dark) photoperiod.

### 2.2 Water deficit experiment

The experiment was conducted in a greenhouse of the Botanic Garden of the University of Coimbra in 2018, from January 24^th^ to May 15^th^. Thirty seedlings from each of the nine selected populations from different mother lineages were individually transplanted into 1L plastic pots (8.6 × 8.6 wide and 21.5 cm deep) filled with a mixture of commercial soil and sand (1:1), resulting in a total of 270 pots. The pots were randomly assigned to a position in the greenhouse bench at the beginning of the experiment and rotated 1-2 times a week throughout the experimental time to account for microclimatic differences at different locations in the bench. Plants were kept at over 80% soil humidity until the beginning of the treatment application. Fifteen pots from each population were randomly assigned to the water deficit treatment, and the remaining 15 were used as control. Plants under the water deficit treatment were kept at 50-40%, and control plants were kept over 80% of field capacity. Soil water content was maintained by weighing the pots every two days and rewetting them to the required water levels. Plants were fertilized twice before the beginning of the water deficit treatment.

### 2.3 Sampling and measurements

Four weeks after the beginning of the water deficit treatment, plants were harvested. Before harvesting, we measured chlorophyll *a* fluorescence and collected leaves for physiological parameters on 10 plants per population and water treatment. Plant biomass was collected, and relative water content, cell membrane permeability, pigments and carbohydrates were analyzed on fresh leaves, and antioxidant activity was analyzed on oven-dried leaves.

#### 2.3.1 Biomass

Plant biomass was separated into above and belowground biomass. Roots were carefully washed to eliminate attached soil particles. Shoots and roots were oven dried at 50 °C until constant weight and weighed.

#### 2.3.2 Chlorophyll a fluorescence and pigments content

Photosynthesis was assessed by measuring the chlorophyll *a* fluorescence using a portable fluorometer (FluorPen FP100 PAM, Photo System Instruments, Czech Republic). After dark adaptation (for at least 30 min), the minimum fluorescence was measured by applying a weak-intensity modulated light and the maximum fluorescence in the dark was measured after using a saturating pulse of light. Then, leaves were adapted to light conditions. The steady-state fluorescence was established, and the maximum fluorescence in light was assessed after a saturating light pulse. The maximum quantum efficiency of photosystem II (*F_v_/F_m_*), the effective quantum efficiency of PSII (Φ_PSII_), the photochemical quenching (qP) and non-photochemical quenching (NPQ) were calculated according to van Kooten and Snel (1990).

Chlorophyll *a* (Chl *a*), chlorophyll *b* (Chl *b*), carotenoids and anthocyanins were quantified as described by Sims and Gamon (2002). For the photosynthetic pigments’ extraction, leaf discs were homogenized with an acetone:50 mM Tris (80:20) buffer, and for the anthocyanins’ extraction, leaf discs were homogenized with a methanol/HCL/H_2_O (90:1:1) solution. After centrifugation (5 000 g for 5 min at 4°C), the absorbance of the acetone extracts was read at 470, 537, 647 and 663 nm and the methanolic extracts were read at 529 and 650 nm using a Jenway 7305 spectrophotometer. The contents of pigments were calculated according to Sims and Gamon (2002).

#### 2.3.3 Cell membrane permeability

Cell membrane permeability (CMP) was determined by electrolyte leakage, as described by Dias et al. (2018). Leaf discs were immersed in de-ionized water and incubated for 24h at room temperature on a rotary shaker. The electrical conductivity was measured before (L_t_) and after (L_0_) samples autoclaving (120°C for 20 min). In addition, the electrolyte leakage (L_t_/L_0_*100) was determined.

#### 2.3.4 Plant water status and carbohydrates contents

Leaf relative water content (RWC) was calculated as RWC (%) = (fresh weight – dry weight)/(turgid weight – dry weight). Briefly, leaves were weighed (fresh weight), placed in 1.5 mL microtubes filled with water, and set overnight in the dark at 4°C. Then, leaves were weighed to obtain the turgid weight, and after drying for 7 days at 40°C, the dry weight was obtained.

Total soluble sugars (TSS) were determined according to Irigoyen et al. (1992) with some modifications. First, leaf discs were homogenized with ethanol at 80% (v/v) and placed in a bath at 80°C for one hour. Then, after centrifugation (5 000*g* for 10 min at 4°C), 30 µl of the supernatant was incubated for 10 min at 100°C with an anthrone solution that contained 40 mg of anthrone, 1 mL of dH_2_O and 20 mL of H_2_SO_4_. After cooling and centrifugation (as described previously), the absorbance of the supernatant was read at 625 nm using a Jenway 7305 spectrophotometer. TSS content was calculated using a glucose standard curve (y=7.197*x* + 0.07, R^2^ = 0.985).

For starch determination, leaf discs were homogenized with perchloric acid (30%, v/v) and incubated at 60°C for one hour (Osaki et al., 1991). After centrifugation (10 000*g* for 10 min at 4°C), the supernatant was incubated with an anthrone solution (as described for TSS) at 100°C for 10 min. Then, the samples were centrifuged (5 000*g*, 10 min, 4°C), and the absorbance was read at 625 nm using a Jenway 7305 spectrophotometer. Starch content was calculated using a glucose standard curve (y=3.84*x* + 0.03, R^2^ = 0.992).

#### 2.3.5 Antioxidant activity

For antioxidant activity (TAA), leaf powder (∼100 mg) was homogenized with methanol and incubated for 30 min at 40°C (Re et al., 1999). After centrifugation (15 000*g*, 15 min at 4°C), the supernatant was mixed with an ABTS (2,20-azino-bis(3-ethylbenzothiazoline-6-sulphonic acid)) solution. The absorbance was read at 734 nm using a Jenway 7305 spectrophotometer. Antioxidant activity was determined using a gallic acid standard curve (y=0.0002*x* + 0.173, R^2^ = 0.984).

### 2.4 Data analysis

To access the effect of genome duplication, we tested the effect of cytotype (diploid, neotetraploid and established tetraploid of var. *maritima* from the contact zone) using a one-way ANOVA. To access the magnitude of response to water deficit of each cytotype, we calculated the response ratio (R, according to Hedges et al. (1999) for every plant growing under water deficit as R = ln(Treatment/Control), where treatment refers to the trait value obtained for the plant under water deficit, and control refers to the mean value of the same trait obtained for the plant cytotype under control conditions. Values closer to zero indicate no response of a given trait to water deficit. Values significantly lower or higher than zero indicate a negative or positive response, respectively, of a given trait to water deficit. One-sample t-test was used to test if the response ratio differed significantly from zero.

To evaluate the performance of cytotypes over the latitudinal gradient, we tested the effect of the population (8 populations distributed along the latitudinal gradient of *J. maritima* distribution range) on the response to water availability of biomass and physiological variables by using one-way ANOVA. Additionally, we tested the effect of variety and cytotype combined (2*x* var. *maritima*, 4*x* var. *maritima* and 4*x* var. *sabularia*) in response to water availability of biomass and physiological variables by using one-way ANOVA. Tukey test was used for *post-hoc* comparisons. Whenever necessary, variables were log-transformed to achieve normality and homoscedasticity. However, for root biomass, normality and homoscedasticity were not fulfilled after transformation; thus, the effect of variety was assessed using the Kruskal-Wallis test with Wilcoxon rank sum for pairwise comparison. The analyses were performed using R software 3.5.2 (R Core development team, 2018) and using the package “car” (Fox and Weisberg, 2019).

## 3 Results

### 3.1 Diploids, neotetraploids and established tetraploids of var. *maritima*

**Figure 1:**
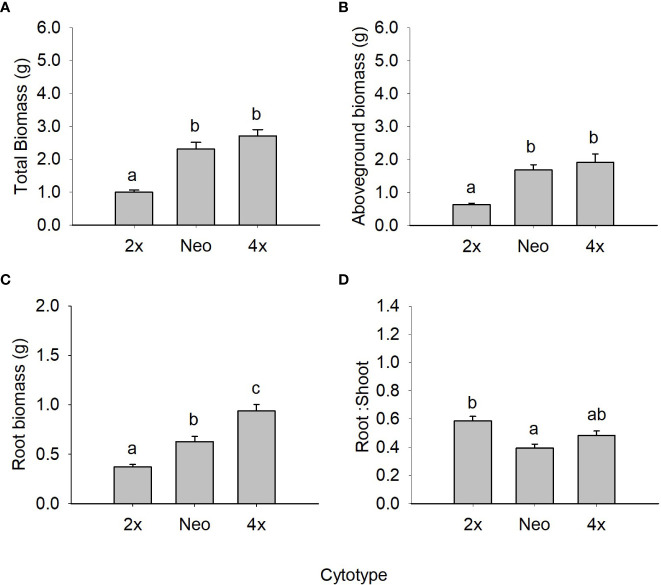
Mean (± SE) for total biomass **(A)**, aboveground biomass **(B)**, root biomass **(C)** and root to shoot ratio **(D)** of diploids, neotetraploids and tetraploids *Jasione maritima* var *maritima* of contact zone populations grown under control conditions. Significant differences at *P* < 0.05 among cytotypes are indicated by different letters.

**Figure 2:**
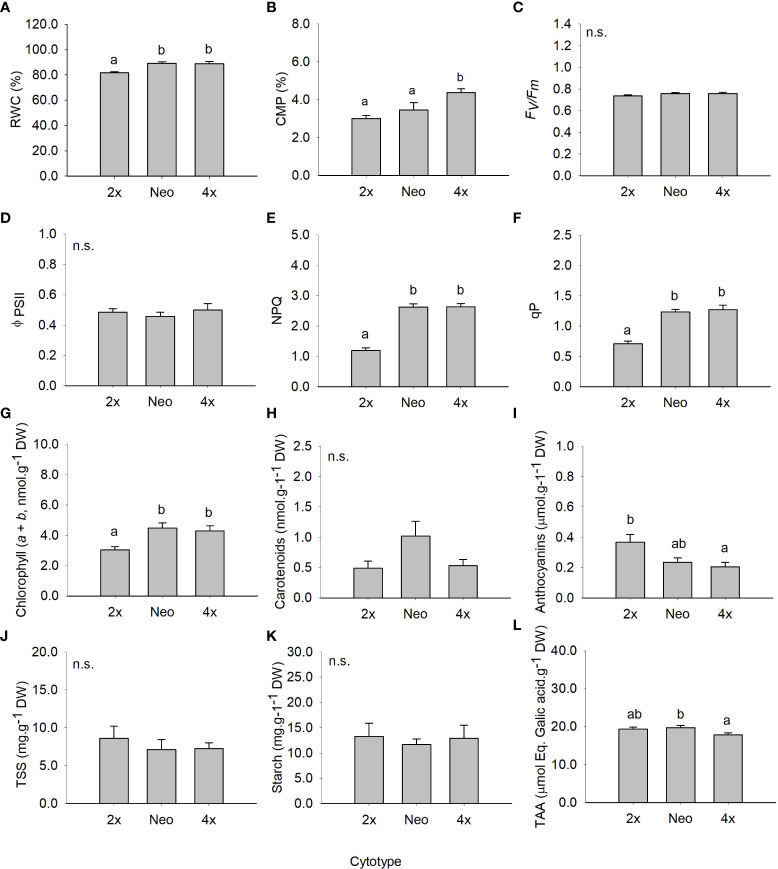
Mean (± SE) for relative water content **(A)**, cell membrane permeability **(B)**, maximum quantum efficiency of photosystem II **(C)**, effective quantum efficiency of PSII **(D)**, non-photochemical quenching **(E)**, photochemical quenching **(F)**, chlorophyll (*a+b*) **(G)**, carotenoid content **(H)**, anthocyanin’s content **(I)**, total soluble sugars **(J)**, starch content **(K)** and total antioxidant capacity **(L)** of diploids, neotetraploids and tetraploids *Jasione maritima* var *maritima* of contact zone populations grown under control conditions. Significant differences at *P* < 0.05 among cytotypes are indicated by different letters. n.s. indicated the absence of significant differences.

When comparing diploids, neotetraploids and tetraploids of var. *maritima* from the contact zone under control conditions, ploidy had a statistically significant effect on all biomass-related traits (**Figure 1**; **Table 2**) and on several physiology-related traits (**Figure 2**; **Table 2**). Diploids had significantly lower total, above and root biomass than neotetraploids and established tetraploids (*P <* 0.05), while the latter two were not different from each other (*P >* 0.05), except for root biomass, where tetraploids showed higher values than neotetraploids (*P <* 0.05; **Figures 1A-C**). Diploids had a significantly higher root-to-shoot biomass ratio than neotetraploids (*P <* 0.05), while tetraploids presented intermediate values (**Figure 1D**).

**Table 2:** Results from the one-way ANOVAs testing for cytotype (Cytotype) effect under control conditions and on the response of morphological and physiological traits under water deficits.

Effect		Cytotype		Cytotype	
		Under control conditions		Response ratio to water deficit	
Response variables	Df	*F*	*P*-values	*F*	*P*-values
Morphological					
Total biomass	2	51.31	**<0.001**	1.99	**0.029**
Aerial biomass	2	49.34	**<0.001**	1.11	0.338
Root biomass	2	41.21	**<0.001**	3.05	0.059
Root : Shoot	2	10.17	**<0.001**	3.79	**0.031**
Physiological					
RWC (%)	2	8.94	**0.001**	0.61	0.550
*F*_v_/*F*_m_	2	1.86	0.175	0.06	0.941
Φ_PSII_	2	0.42	0.663	3.05	0.066
NPQ	2	67.70	**<0.001**	0.09	0.913
qP	2	31.87	**<0.001**	2.92	0.071
CMP	2	18.84	**<0.001**	12.62	**<0.001**
Chl (*a*+*b*)	2	6.61	**0.005**	0.82	0.450
Anthocyanins	2	4.96	**0.015**	0.42	0.662
Carotenoids	2	1.99	0.160	0.64	0.535
TSS	2	0.413	0.666	1.86	0.176
Starch	2	0.009	0.991	0.56	0.580
TAA	2	3.47	**0.048**	1.04	0.367

Significant P values (P < 0.05) are highlighted in bold.

Leaf relative water content (RWC), NPQ, qP and chlorophyll (*a* + *b*) were higher in neotetraploids and established tetraploids than in diploids (*P <* 0.05; **Figures 2A, E-G, L**). Cell membrane permeability (CMP) was higher in established tetraploids compared to diploids and neotetraploids (*P <* 0.05; **Figure 2B**). Anthocyanins’ contents were significantly higher in diploids compared to established tetraploids (*P <* 0.05) with differences between diploids and neotetraploids being marginally non-significant (*P* = 0.064; **Figure 2I**). TAA was higher in neotetraploids compared to established tetraploids (*P <* 0.05) with diploids having intermediate values (**Figure 2K**). Photosynthesis related parameters *F*’v/F’_m_ and Φ_PSII_, carotenoids’ content, TSS and starch were not significantly affected by ploidy level (**Figures 2C, D, H, J, K**; **Table 2**).

### 3.2 Response to water deficit of var. *maritima* cytotypes

**Figure 3:**
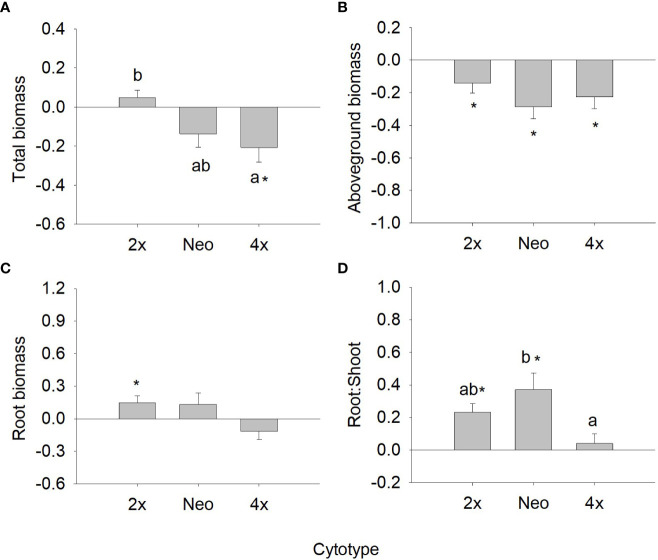
Response ratio (mean ± SE) for total biomass **(A)**, aboveground biomass **(B)**, root biomass **(C)** and root to shoot ratio **(D)** of diploids, neotetraploids and tetraploids *Jasione maritima* var *maritima* of contact zone populations. Significant differences at *P* < 0.05 among cytotypes are indicated by different letters. The presence of * indicates that the response ratio differed significantly from zero.

When comparing the response ratio of diploids, neotetraploids and tetraploids of var. *maritima* to water defict, ploidy had a statistically significant effect on total biomass, root:shoot ratio and CMP (**Table 2**), and a marginally non-significant effect for root biomass (*P =* 0.059), Φ_PSII_ (*P =* 0.066) and qP (*P =* 0.071). For total biomass, diploids had significantly higher response ratios than tetraploids (*P <* 0.05), while neotetraploids presented intermediate values (*P >* 0.05) (**Figure 3A**). For root:shoot ratio, neotetraploids had significantly higher response ratios than tetraploids (*P <* 0.05), while diploids presented intermediate values (*P >* 0.05) (**Figure 3D**). Lastly, for CMP, diploids and neotetraploids behaved similarly (*P >* 0.05) and had significantly higher response ratios than tetraploids (*P <* 0.05) (**Figure 3A**). No statistically significant differences were observed for the remaining variables (**Table 2**).

**Figure 4:**
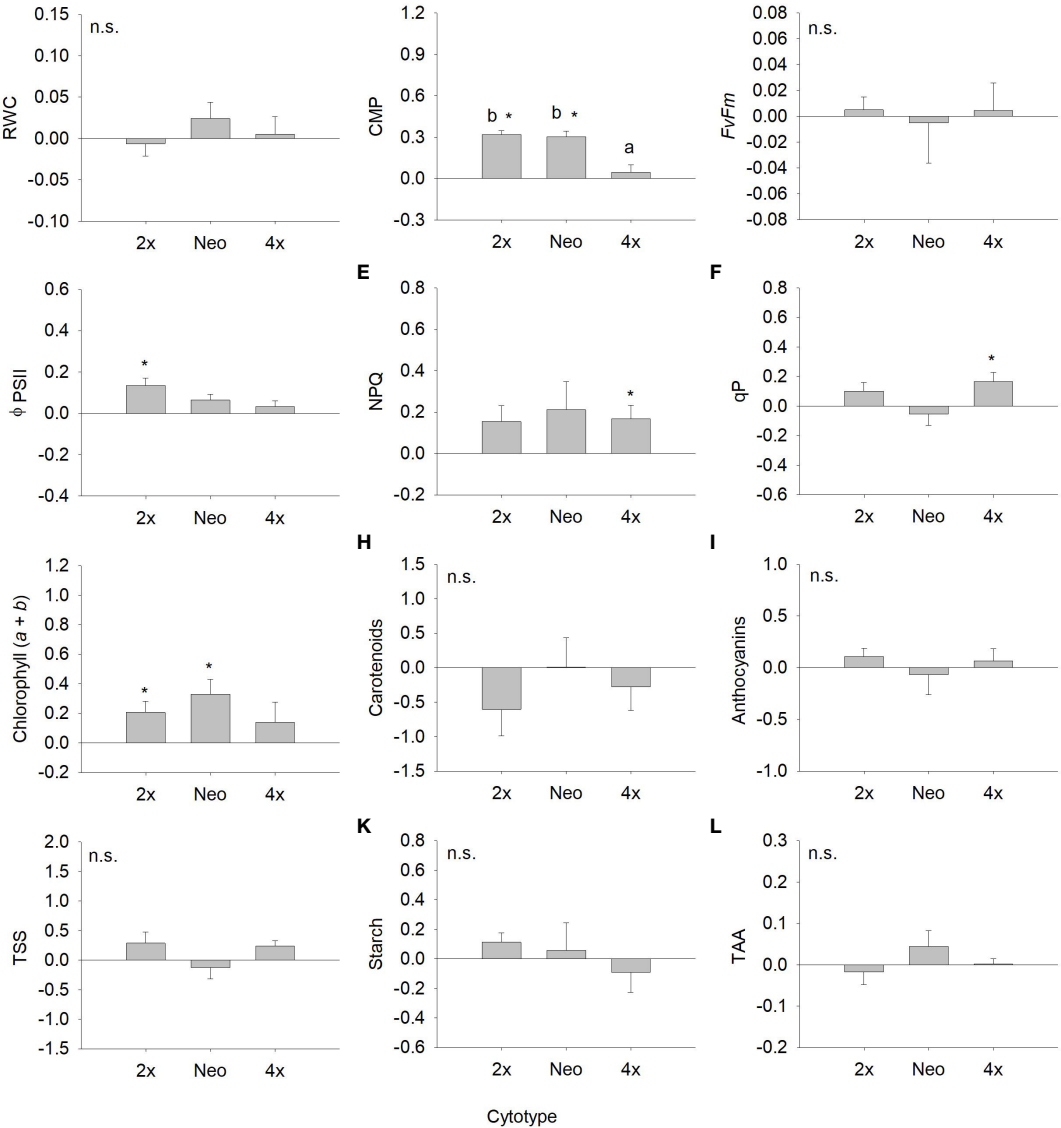
Response ratio (mean ± SE) for relative water content **(A)**, cell membrane permeability **(B)**, maximum quantum efficiency of photosystem II **(C)**, effective quantum efficiency of PSII **(D)**, non-photochemical quenching **(E)**, photochemical quenching **(F)**, chlorophyll (*a+b*) **(G)**, carotenoid content **(H)**, anthocyanin’s content **(I)**, total soluble sugars **(J)**, starch content **(K)** and total antioxidant capacity **(L)** of diploids, neotetraploids and tetraploids *Jasione maritima* var *maritima* of contact zone populations. Significant differences at *P* < 0.05 among cytotypes are indicated by different letters. The presence of * indicates that the response ratio differed significantly from zero.

Analysing the strength of the response assessing differences in the response ratios from zero, differences were observed for several traits, although variable among cytotypes (**Figure 3**, **4**). For total biomass, diploids had positive responses to water deficit and neotetraploids and tetraploids had negative responses, but only the latter differed significantly from zero (*P <* 0.05; **Figure 3A**). Aboveground biomass showed a significant and negative response to water deficit for the three cytotypes (*P <* 0.05; **Figure 3B**). The response of root biomass was positive for diploids (*P <* 0.05) and, although not significant, for neotetraploids (*P >* 0.05), and marginally negative in tetraploids (*P* = 0.085; **Figure 3C**). The response of root:shoot ratio was significantly positive for diploids and neotetraploids (*P <* 0.05), while no impact was detected in tetraploids (**Figure 3D**). The impact of water deficit on physiology-related traits was weaker. Diploids had significantly positive responses for Φ_PSII_ and chlorophyll (*a* + *b*), neotetraploids had significantly positive responses for chlorophyll (*a* + *b*), and tetraploids had significantly positive responses for NPQ and qP (**Figures 4D-G**). RWC, *F*’v/F’_m_, carotenoid and anthocyanins’ contents, TSS, Starch and TAA did not respond significantly to water deficit (**Figures 4A, C, H-L**).

### 3.3 Response to water deficit across the latitudinal gradient

When analyzing all the populations included in the study individually, we did not observe a defined response pattern along the latitudinal gradient of the distributional range of the *Jasione maritima* complex (Appendix 1 – **Figures 1A-D**). For some variables, we found significant differences among populations in the magnitude of the response (*e.g.*, cell membrane permeability, qP, Chlorophyll (*a + b*), TAA (Appendix 1 - **Figures 2B, F, G, I, L**)), but these did not follow a pattern; instead, results are population dependent.

**Figure 5:**
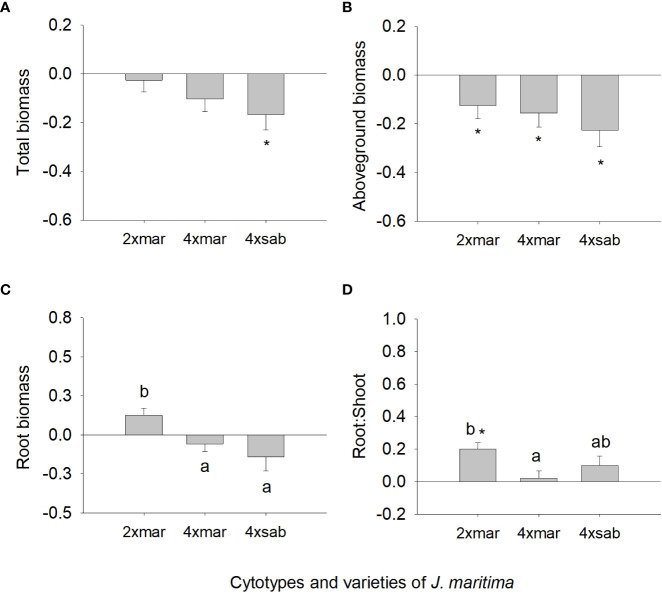
Response ratio (mean ± SE) for total biomass **(A)**, aboveground biomass **(B)**, root biomass **(C)** and root to shoot ratio **(D)** of populations of diploid and tetraploid *Jasione maritima* var *maritima* and tetraploid *Jasione maritima* var *sabularia.* Significant differences at *P* < 0.05 among cytotypes are indicated by different letters. The presence of * indicates that the response ratio differed significantly from zero.

**Figure 6:**
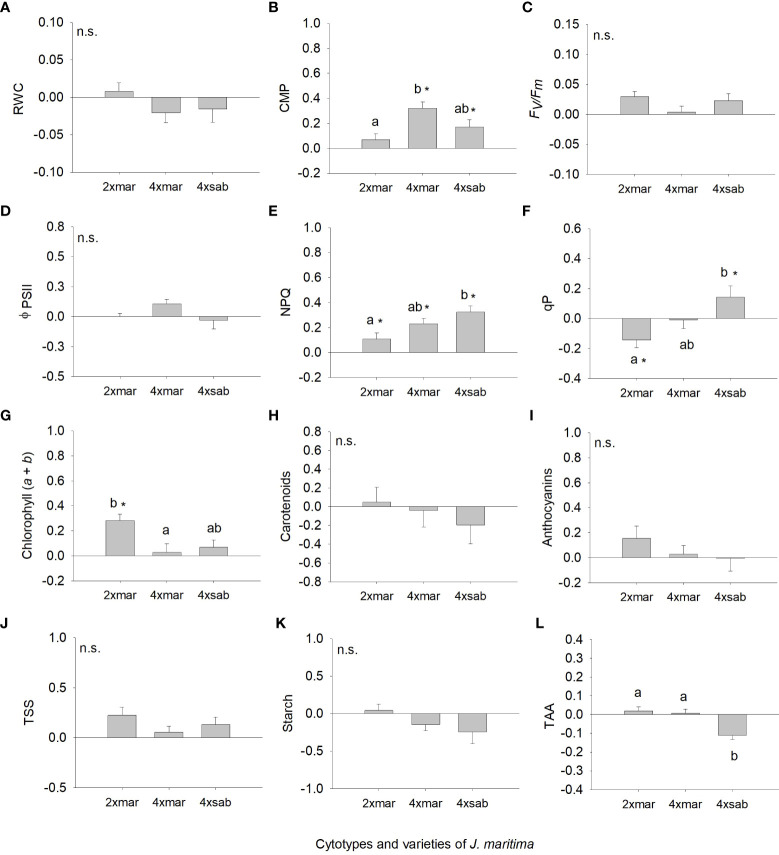
Response ratio (mean ± SE) for relative water content **(A)**, cell membrane permeability **(B)**, maximum quantum efficiency of photosystem II **(C)**, effective quantum efficiency of PSII **(D)**, non-photochemical quenching **(E)**, photochemical quenching **(F)**, chlorophyll (*a+b*) **(G)**, carotenoid content **(H)**, anthocyanin’s content **(I)**, total soluble sugars **(J)**, starch content **(K)** and total antioxidant capacity **(L)** of populations of diploid and tetraploid *Jasione maritima* var *maritima* and tetraploid *Jasione maritima* var *sabularia.* Significant differences at *P* < 0.05 among cytotypes are indicated by different letters. The presence of * indicates that the response ratio differed significantly from zero. n.s. indicates the absence of significant differences.

However, we found differences among *J. maritima* entities, i.e., diploid var. *maritima*, tetraploid var. *maritima* and tetraploid var. *sabularia*, for root biomass, root:shoot ratio, NPQ, qP, CMP, chlorophyll (*a* + *b*) and TAA (**Table 3**). Diploid var. *maritima* had significantly higher root biomass response ratios than that of tetraploid varieties (**Figure 5C**). The response of root:shoot ratio and chlorophyll (*a* + *b*) was significantly higher in diploid var. *maritima* than in tetraploid var. *maritima*, with tetraploid var. *sabularia* having intermediate values (**Figure 5D**, **6G**). Tetraploid var. *maritima* had significantly higher CMP response ratios than diploid var. *maritima*, and tetraploid var. *sabularia* had intermediate values (**Figure 6B**). For NPQ and qP, tetraploid var. *sabularia* had significantly higher response ratios than diploid var. *maritima*, and tetraploid var. *maritima* had intermediate values (**Figures 6E, F**). Finally, both cytotypes of var. *maritima* had significantly higher TAA response ratios than tetraploid var. *sabularia* (**Figure 6L**). No differences between *J. maritima* entities were observed in the remaining variables (**Table 3**).

**Table 3:** Results from the one-way ANOVA or Kruskal-Wallis testing for the effect of variety (2*x* var. *maritima*, 4*x* var. *maritima* and 4*x* var. *sabularia*) on the response of morphological and physiological traits measured.

Effect	Variety		
Response variables	df	*F* or *χ^2^*	*P*-values
Physiological			
RWC (%)	2	0.81	0.447
*F*_v_/*F*_m_	2	2.00	0.142
Φ_PSII_	2	4.55	0.103
NPQ	2	**4.83**	**0.011**
qP	2	**5.51**	**0.006**
CMP	2	**9.146**	**0.010**
Chl (*a*+*b*)	2	**5.38**	**0.007**
Anthocyanins	2	0.86	0.428
Carotenoids	2	0.45	0.640
Sugars	2	1.49	0.232
Starch	2	1.97	0.146
TAA	2	**8.60**	**< 0.001**
Morphological			
Total biomass	2	1.56	0.215
Aboveground biomass	2	0.64	0.532
Belowground biomass	2	**8.91**	**0.012**
Root : Shoot	2	**4.23**	**0.017**

Significant P values (P < 0.05) are highlighted in bold.

When analysing differences from zero in response ratios, differences were observed for total biomass, aboveground biomass, root:shoot ratio (**Figure 5**), CMP, NPQ, qP and chlorophyll (*a* + *b*) (**Figure 6**). Diploid var. *maritima* has significantly negative response ratios for aboveground biomass and qP, and significantly positive response ratios for root:shoot, NPQ and chlorophyll (*a* + *b*) (*P <* 0.05). Tetraploid var. *maritima* has significantly negative response ratios for aboveground biomass, and significantly positive response ratios for CMP and NPQ (*P <* 0.05). Finally, tetraploid var. *sabularia* has significantly negative response ratios for total and aboveground biomass, and significantly positive response ratios for CMP, NPQ and qP (*P <* 0.05).

## 4 Discussion

Overall, our results show evidence of the effect of whole genome duplications on plant biomass and some physiology-related traits that could confer some benefit to tetraploids under water deficit conditions. In the contact zone, diploids seem to use a strategy of avoidance. In contrast, tetraploids seem to employ a strategy of tolerance to overcome water stress, which appears to be equally efficient as we did not find significant differences between cytotypes in aboveground biomass production and traits related to efficiency in response to water deficit. The response of neotetraploids to water deficit was similar to that of diploids suggesting that the higher tolerance of tetraploids to water stress is not a direct effect of genome duplications. We did not observe a defined response pattern along the latitudinal gradient of the distributional range of the *J. maritima* complex, indicating that the response to water deficit was population dependent. These results are expected to impact the distribution of cytotype and are discussed below in more detail.

### 4.1 Do var. *maritima* diploids, neotetraploids and established tetraploids differ in morphological and physiological traits?

We hypothesized that genome duplications produce changes in the newly arisen var. *maritima* tetraploids; if so, neotetraploids are expected to differ from diploids and advantageous novel traits can be maintained and selected. Our data shows that biomass, chlorophyll content, RWC, NPQ and qP in neotetraploids and established tetraploids of var. *maritima* differ from diploids and that these differences could be attributed to genome duplications. Polyploidization increased chlorophyll’s content, which suggests that neotetraploids and established tetraploids have a higher capacity for light absorption than diploids (Cao et al., 2018). A similar response to polyploidization was reported for *Acacia mearnsii* (Mathura, 2006), *Populus* (Liao et al., 2016), *Chrysanthemum nankingens* (Dong et al., 2017) and *Lilium* FO hybrids (Cao et al., 2018). Higher chlorophyll contents in polyploids were associated with an upregulation of genes related to chlorophyll synthesis and/or downregulation of chlorophyll degradation genes (Dong et al., 2017). Additionally, tetraploids of var. *maritima* also showed higher qP, indicating that they have a higher proportion of PSII reaction centres open (Murchie and Lawson, 2013). Nevertheless, the other two typical chlorophyll fluorescence parameters (F_v_/F_m_ and Φ_PSII_) commonly used to assess the relative state of photosystem II showed no differences among the three cytotypes, indicating that they have a similar efficiency of photochemistry (Maxwell and Johnson, 2000). These findings align with other studies that compared photosynthetic efficiency between diploids and tetraploids (Jaskani et al., 2005; Vyas et al., 2007; Mao et al., 2018).

Despite the similar photosynthetic efficiency (which may represent similar availability of NADPH and ATP for the Calvin cycle), var. *maritima* diploids and tetraploids differed in biomass production. Compared to diploids, the higher biomass production in neotetraploids and established tetraploids var. *maritima* may be related to improved light-independent photosynthesis reactions (Calvin cycle) as demonstrated for other polyploids [*e.g.*, *Populus* (Liao et al., 2016), *Robinia pseudoacacia* (Wang et al., 2013)]. Polyploidization improves the CO_2_ assimilation rate per leaf area and increases the expression and activity of RuBisCO and other enzymes involved in the Calvin Cycle (Vyas et al., 2007; Liao et al., 2016) with the consequent increase in carbohydrate production. Our data shows that var. *maritima* tetraploids can efficiently use carbohydrates to produce more biomass and even maintain basal levels of soluble sugars and reserve sugars (starch) similar to diploids. Additionally, polyploids often have leaves with larger stomata and mesophyll cells and more chloroplasts and chlorophyll than content diploids, which strongly influence gas exchange, ultimately promoting higher plant growth (Del Pozo and Ramirez-Parra, 2014). Indeed, stomata density was significantly higher in diploids than in the neotetraploids of *J. maritima*, while the stomata length of neotetraploids was considerably higher than that of diploids (Siopa et al., 2020). The morpho-anatomical differences between diploids and tetraploids (*e.g.*, cell size, xylem vessels, hydraulic conductivity, stomatal density and size) also affect the water movement in the continuum soil–plant-atmosphere (Hao et al., 2013; Del Pozo and Ramirez-Parra, 2014; Siopa et al., 2020; Walczyk and Hersch-Green, 2022). In polyploids, the larger stomata (with lower stomatal aperture) and the lower stomata density contribute to decreased transpiration rates and higher water availability (van Laere et al., 2011; Del Pozo and Ramirez-Parra, 2014; Siopa et al., 2020). Consistent with this, if, on the one hand, our results for RWC show that var. *maritima* neotetraploids and tetraploids have higher RWC compared to diploids, on the other hand, diploids invested proportionally more on root biomass (higher root:shoot) than polyploids, possibly as a compensation mechanism to maintain the water levels.

Cell membrane permeability is widely used as a biomarker of oxidative stress (Demidchik et al., 2014) and, in this study, established tetraploids had higher CMP than diploids and neotetraploids, suggesting that this attribute was not affected by genome duplications. Furthermore, polyploidization has been referred to improve the plant antioxidant system, increasing the tolerance of polyploids to stress conditions (Zhang et al., 2010; Wang et al., 2013; Tan et al., 2015; Kong et al., 2017). However, TAA levels did not differ between var. *maritima* diploids and tetraploids, suggesting that to deal with this high CMP, tetraploid plants possibly use other protective mechanisms, such as the “primary” antioxidant system (Agati et al., 2012), maintaining, for example, high levels of the antioxidant enzymes, ascorbate and glutathione to balance the oxidative status (Zhang et al., 2010; Wang et al., 2013; Mao et al., 2018). This hypothesis is supported by the higher biomass production observed in var. *maritima* tetraploids, reinforcing its capacity to sustain higher levels of growth, despite cell membrane injuries.

### 4.2 Do differences between var. *maritima* cytotypes increase the ability to cope with exposure to water deficit?

Our results show differences in the response of diploids and tetraploids var. *maritima* to water deficit for several traits, suggesting different abilities to cope with drought stress. However, the response of diploids and tetraploids under water deficit conditions differed significantly only for CMP, total biomass and root:shoot. Tetraploids showed higher basal levels of membrane injury (high CMP) under control conditions compared to diploids, which may provide benefits under stress, promoting a more efficient antioxidant response that helps to maintain membrane damage under stress conditions. Under water deficit conditions, CMP response was higher in diploids and neotetraploids than in established tetraploids, suggesting the occurrence of membrane damage (Bajji et al., 2002; Farooq and Azam, 2006). More significant membrane damage in diploids, when compared to tetraploids, was also reported for other species under water deficit [e.g., *Citrus wilsonii* (Jiang et al., 2022), *Poncirus trifoliata* (Wei et al., 2019)] and may result from an excessive increase of reactive oxygen species (ROS; (Wang et al., 2013; Wei et al., 2019). Overall, diploids and neotetraploids showed higher membrane damage in response to water stress, but they also invested more in root than shoot growth in response to water deficit than tetraploids. Investing proportionally more in root growth is a strategy to avoid water deficit by increasing water acquisition (Farooq et al., 2009; Poorter et al., 2012). Diploids and neotetraploids may thus compensate for their lower tolerance to water deficit by avoiding water stress (*e.g.*, increasing water uptake). This strategy may have allowed them to keep similar levels of photosynthesis and aboveground biomass production under water deficit, likely, by activating biochemical (*e.g.*, more efficient antioxidant response) and/or morphological changes (*e.g.*, lower stomatal density) to protect cells from injury (as described above). Interestingly, this is in accordance with niche modelling results that suggest that current distribution of diploids is restricted to a smaller area than its potential suitable area, and that developmental and physiologic traits do not constrain them to colonise drier southern regions (Castro et al., 2020).

Unlike the reported in some studies (*e.g.*, Pavlíková et al., 2017; Martínez et al., 2018), the neotetraploids and established tetraploids did not perform better under water deficit conditions than diploids as indicated by negative responses in total biomass production and lack of differences in the F_v_/F_m_ and Φ_PSII_. Similar results were found by (Walczyk and Hersch-Green, 2022) for diploid and tetraploid *Solidago gigantea*, in which cytotypes did not differ in biomass and photosynthetic efficiency in response to water deficit. Regardless of whether the ecological differences arose in association with or after genome duplications, niche differentiation has been suggested as a key factor for the successful establishment of polyploids. However, our data does not support that this might have been the case in var. *maritima* tetraploid establishment. Neotetraploids, even if presenting the highest trade-off between above and belowground biomass to compensate for the injuries caused by water deficit stress, had a capacity to colonise dry environments areas similar to that of diploids. Thus, neotetraploids may have emerged in the driest peripheral areas of the diploids distribution range, and selective pressures (together with other processes) may have selected individuals with more drought tolerance (lower CMP response), enabling them to expand their distribution further south.

It is also important to note that, despite the substantial reduction in soil water percentage (moderate water deficit, following Yuan et al., 2016), the water deficit treatment did not induce adverse effects on cell hydration (no changes in RWC). This suggests that the moderate water deficit applied was insufficient to impact the water status of this species. *Jasione maritima* grows in the dune system, where the soil drains rapidly, and the plants are exposed to harsh conditions. Further studies with reduced soil field capacity and severe water deficit (below 30%) during a more extended period may be necessary to obtain a more robust response.

### 4.3 Does response to water deficit of *Jasione maritima* varieties and cytotypes vary across a latitudinal gradient?

We hypothesized that tetraploids have higher drought tolerance and consequently higher fitness under stressful conditions than diploids explaining the current distributional patterns where tetraploids occupy southern locations marked by higher temperatures and more extended drought periods, while diploids occupy northern locations with higher moisture environmental values. Our results show no defined response pattern to water deficit along the latitudinal gradient of the distributional range of the *J. maritima* complex and this is consistent with the results obtained at the contact zone (discussed above). Yet, overall, the cytotypes differed in their response to water deficit conditions. Diploid populations invested comparatively more in root biomass than shoot biomass in response to water deficit, as indicated by the significant differences in the response patterns in root biomass in diploids compared to tetraploids. Although we did not find statistically significant results for RWC, there was a tendency for a positive response in diploids and a negative response in tetraploids. These results suggest a strategy by diploids of var. *maritima* to avoid water deficit by increasing water acquisition (investment in root), as mentioned above.

Tetraploids of var. *sabularia* showed an increased response in NPQ under water deficit conditions, suggesting a higher ability of the tetraploids var. *sabularia* to dissipate excess energy, compared to diploids, thereby protecting the photosystem from possible damages (Pintó-Marijuan and Munné-Bosch, 2014). Despite this, tetraploids of var. *sabularia* had the lowest biomass production values in response to water deficit. Furthermore, these populations also showed the lowest values of TAA and carotenoid content in response to water deficit, suggesting a lower ability to cope with oxidative stress, which may have contributed to a lower ability to sustain biomass production.

Tetraploid var. *maritima* showed intermediate biomass production values in response to water deficit. They showed the most vigorous response in CMP, suggesting the occurrence of membrane damage (Bajji et al., 2002; Farooq and Azam, 2006). This contrasts with the results from the contact zone, where tetraploid var. *maritima* showed lower damage than the diploid counterpart. The population-dependent responses in our study may explain this. Both population SC73 (diploid from contact zone) and population SC72 (tetraploid from contact zone) contrast in CMP response when compared to the other populations from the same ploidy in this study (Appendix - **Figure 2**). Population-dependent results have been reported in other studies. For example, Martínez et al. (2018) showed that distinct Iberian populations of the allotetraploid *Brachypodium hybridum* had different physiologic responses to water stress.

Overall, and despite the lack of significant differences in many cases, diploid populations of *J. maritima* var. *maritima* were less affected by water deficit as they showed the lowest reduction in biomass production. This was greatly due to a more substantial investment in root biomass. Maximising water uptake and avoiding tissue desiccation may benefit diploids over tetraploids under water stress conditions, but further studies are necessary to understand better the response of different populations and ploidies of *J. maritima* complex to environmentally stressful conditions.

## 5 Conclusions

Overall, our results support that genome duplications have produced developmental and physiological changes in neotetraploids (and established tetraploids) of var. *maritima*. In response to the water deficit, diploids seem to use a strategy of avoidance, whereas tetraploids seem to employ the strategy of tolerance to overcome water deficit stress, which appears equally efficient. Contrary to our hypothesis, tetraploids were not more tolerant to water deficit, nor could differences between diploids and tetraploids explain the current distributional patterns of the *J. maritima* complex. First, the results show no defined response pattern to water deficits along the latitudinal gradient of the distributional range of the *J. maritima* complex; instead, they indicate population-dependent results. Further studies are necessary to understand the role of ploidy in explaining the distribution patterns of the *J. maritima* complex.

## Data availability statement

The raw data supporting the conclusions of this article will be made available by the authors, without undue reservation.

## Author contributions

HC was responsible for experimental work, data collection, data analysis and leading manuscript preparation. MD participated in data collection and manuscript preparation. MC participated in data collection and manuscript preparation. JL participated in manuscript preparation and funding acquisition. SC participated in data collection, manuscript preparation and funding acquisition. All authors contributed to the article and approved the submitted version.

## Supplementary material

Ensure that any supplementary material is correctly published at this link: https://www.frontiersin.org/articles/10.3389/fpls.2023.1144678/full#supplementary-material
