# Supplementary material for: Impact of genome duplications in drought tolerance and distribution of the diploid-tetraploid Jasione maritima
Source: Front Plant Sci. 2023 Feb 23;14:1144678. doi: 10.3389/fpls.2023.1144678 (PMC9995889; doi:10.3389/fpls.2023.1144678)
Supplement: Supplementary file 1 [file DataSheet_1.docx]

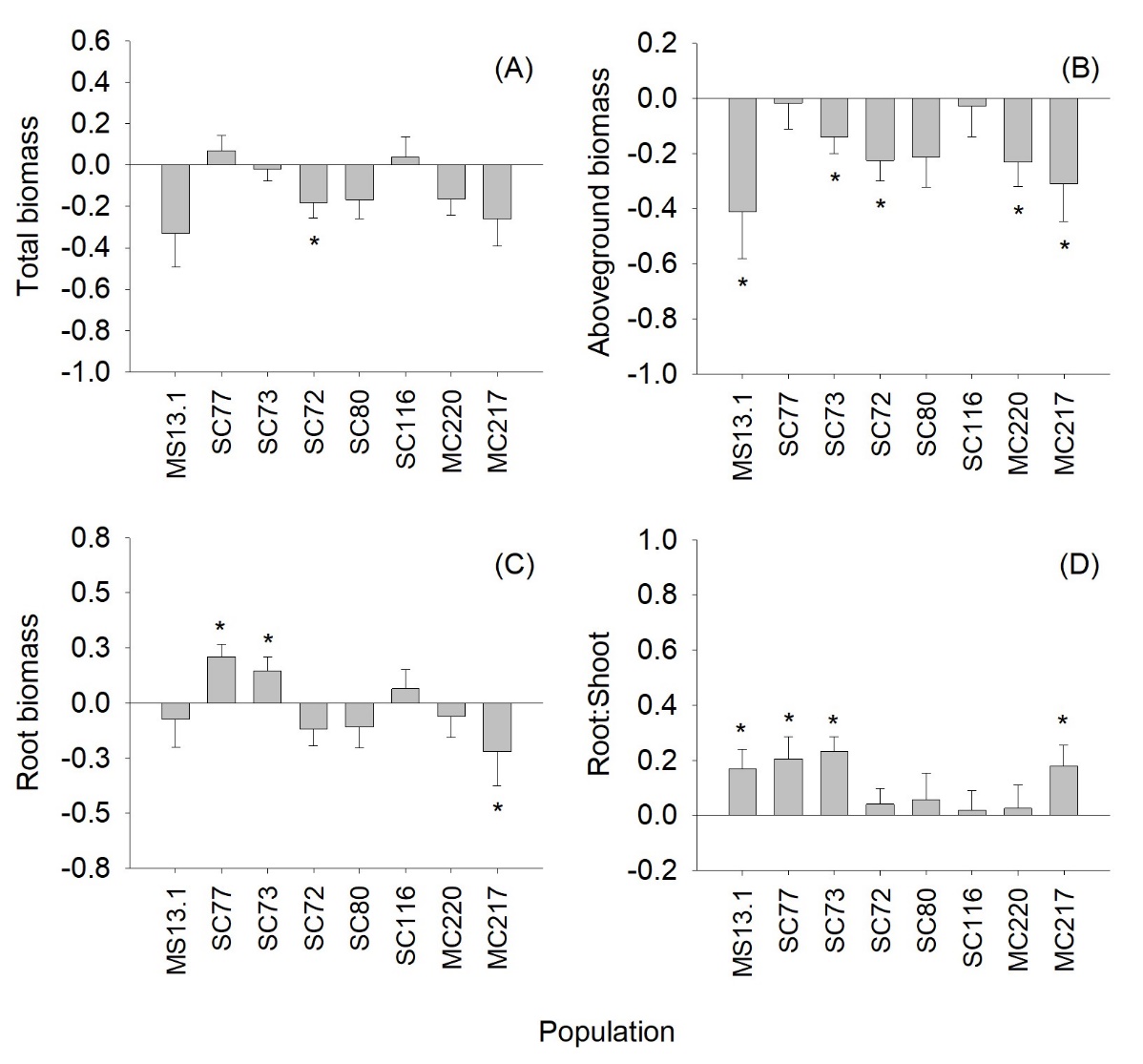


Figure 1. Response ratio (mean ± SE) for total biomass (A), above ground biomass (B), root biomass (C) and root to shoot ratio (D) of each population of diploid and tetraploid *Jasione maritima* var *maritima* and tetraploid *Jasione maritima* var *sabularia.* Significant differences at p < 0.05 among cytotypes are indicated by different letters. The presence of * indicates that response ratio differed significantly from zero.


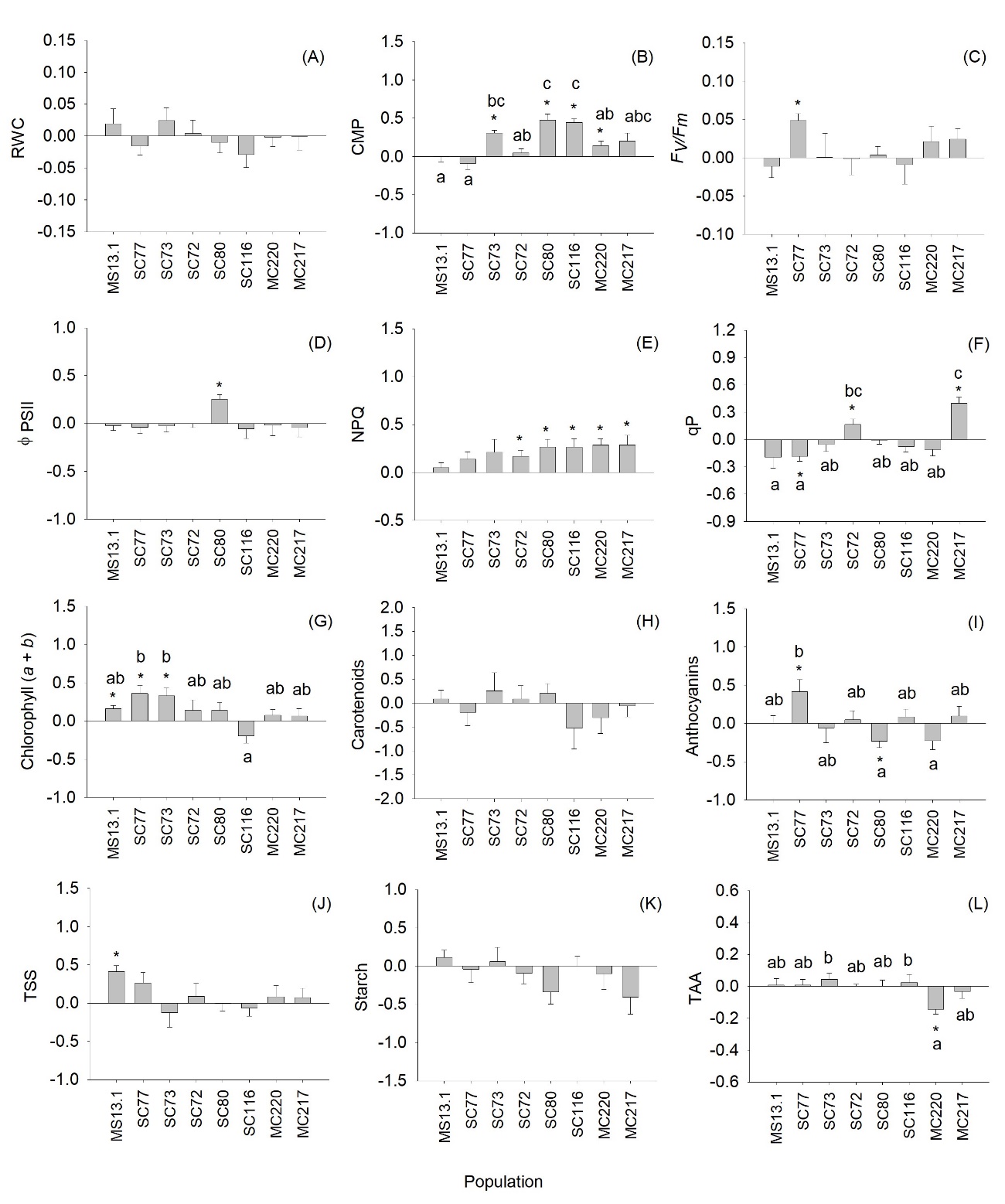


Figure 2. Response ratio (mean ± SE) for relative water content (A), cell membrane permeability (B), maximum quantum efficiency of photosystem II (C), effective quantum efficiency of PSII (D), non-photochemical quenching (E), photochemical quenching (F), chlorophyll (a+b) (G), carotenoid content (H), anthocyanin’s content (I), total soluble sugars (J), starch content (K) and total antioxidant capacity (L) of each population of diploid and tetraploid *Jasione maritima* var *maritima* and tetraploid *Jasione maritima* var *sabularia.* Significant differences at p < 0.05 among cytotypes are indicated by different letters. The presence of * indicates that response ratio differed significantly from zero.
